# The association between unilateral and bilateral performance-related measures in elite female soccer players: a multifaceted investigation

**DOI:** 10.3389/fphys.2024.1298159

**Published:** 2024-06-17

**Authors:** Armin Huso Paravlic, Ensar Abazovic, Zoran Milanović, Goran Vučković, Darjan Spudić, Vedran Hadzic, Maja Pajek, Janez Vodičar

**Affiliations:** ^1^ Faculty of Sport, University of Ljubljana, Ljubljana, Slovenia; ^2^ Science and Research Centre Koper, Institute for Kinesiology Research, Koper, Slovenia; ^3^ Faculty of Sports Studies, Masaryk University, Brno, Czechia; ^4^ Faculty of Sport and Physical Education, University of Sarajevo, Sarajevo, Bosnia and Herzegovina; ^5^ Faculty of Sport and Physical Education, University of Nis, Nis, Serbia

**Keywords:** TMG, neuromuscular function, physical function, strength, women’s football, power, running speed and agility, muscle contractile properties

## Abstract

**Purpose:**

The present study aimed to investigate a) the associations between bilateral performance utilizing countermovement jump (CMJ), squat jump (SJ), speed and unilateral CMJ, isokinetic peak torque in knee extension and flexion with angular velocities of 60°/s and 180°/s and tensiomyography (TMG) parameters; b) whether the asymmetries derived from unilateral tests are associated with bilateral CMJ, SJ and speed in elite female soccer players.

**Methods:**

Thirty-five elite female soccer players (average age: 20 ± 5 years) completed CMJ, SJ, speed, isokinetic muscle strength and TMG tests.

**Results:**

Compared to the non-dominant leg, the dominant leg demonstrated greater peak torque output in both knee flexion (7.4%) and knee extension (5.6%) isokinetic tasks, as well as m. vastus medialis contraction time (7.6%), and soccer-specific agility test (4.1%). Conversely, the hamstring to quadriceps peak torque ratio at 180°/s (8.5%) was significantly greater in the non-dominant leg. The associations between CMJ, SJ and speed performance were positive and ranged from weak (*r* = 0.350) to high (*r* = 0.710). For speed and TMG-derived variables, correlations were negative and ranged from weak (*r* = −0.345, *p* = 0.042, for vastus medialis contraction time) to moderate (*r* = −0.530, *p* = 0.001, for biceps femoris contraction time). Furthermore, both bilateral CMJ and SJ negatively correlated with TMG-derived variables, ranging from weak (*r* = −0.350, *p* = 0.039, for vastus lateralis contraction time) to moderate (*r* = −0.537, *p* = 0.003, for rectus femoris contraction time).

**Conclusion:**

The overall significant, albeit inconsistent, correlations between the diverse performance scores obtained highlight the necessity for a multifaceted and thorough diagnostic strategy in female soccer players.

## 1 Introduction

Muscle power is an important factor in successful rapid movement performance like jumping, sprinting, and kicking (Newton and Kraemer, 1994). Such movements generally require optimal inter-limb balance (Bishop et al., 2018), regardless of lateral preference. Previous authors found >10% asymmetry in peak power tended to decrease jumping ability (Bell et al., 2014). Lateral preference, common among the vast majority of individuals (Annett, 1972), and repeated unilateral sporting activities can lead to muscle asymmetry (Heil et al., 2020). In recent years, an increasing number of studies have examined the relationship between muscle characteristics and their asymmetries and also between sprint and jumping ability (Gil et al., 2015; Bishop et al., 2021; Loturco et al., 2019; Bishop, Coratella, et al., 2021).

Many authors have sought to determine different muscle characteristics and the relationship between different testing methods but have come to different conclusions. For example, several studies (Binet et al., 2005; Almuzaini and Fleck, 2008; Križaj et al., 2019) found weak to high significant positive correlation coefficients (ranging 0.31–0.71) between isokinetic knee extension peak torque and vertical jump performance whilst others reported none (Atabek et al., 2009) or weak insignificant negative correlation coefficients (−0.12) (Maly et al., 2013). Similar discrepancies are present in studies using tensiomyography (TMG) with the same objective (Gil et al., 2015; Loturco et al., 2018; Pereira et al., 2018; Lewis et al., 2022). TMG is a recent method for evaluating the contractile properties of superficial skeletal muscles (Paravlić et al., 2017). It assesses radial muscle deformation caused by electrical stimuli (Paravlić et al., 2020) and provides valuable parameters related to muscle function and structure (Macgregor et al., 2018). One of the mostly reported and clinically important TMG-derived variables are presented through the time of contraction (Tc) and displacement measure (Dm) (Pišot et al., 2008; Šimunic et al., 2011; Paravlić et al., 2022). In contrast to established methods like isokinetic dynamometry and force plates, TMG’s neuromuscular assessment is not influenced by factors like motivation or voluntary effort, which can influence athletic performance (Macgregor et al., 2018; Paravlic et al., 2022). For example, Loturco et al., 2015 and Pereira et al., 2018 found a significant moderate negative correlation between TMG parameters and vertical jump performance. In contrary, Gil et al., 2015 and Lewis et al., 2022 found non-significant correlation between aforementioned variables. These variations may be due to differences in the population evaluated, the TMG measurement technique including measuring point and the electrode positioning, and, most likely, evaluated muscles. Consequently, Loturco et al., 2015 claimed that TMG can discriminate endurance and power athletes whereas Lewis et al., 2022 state the opposite. Consequently, unclear findings in team sports and limited evidence in female soccer players emphasize a need for further investigation in this area.

Although the investigation of inter-limb asymmetry is not a new concept in sports science, researchers have recently intensified their efforts to explore the relationship between asymmetries and athletic performance. A systematic review (Bishop et al., 2018) reported inconsistent findings regarding asymmetry prevalence and its effects on athletic performance. Nevertheless, the literature as a whole indicates there is a high prevalence of muscle asymmetry. This has been measured and found using sports specific tasks (Lockie et al., 2014), dynamometry (Križaj et al., 2019), tensiomyography (Gil et al., 2015), anthropometry (Trivers et al., 2014), in general population (Maloney, 2019) and in the physically active individuals (Lockie et al., 2014).

Athletes in team sports such as soccer primarily use their dominant leg for technical actions like ball control, dribbling, passing, and shooting. Consequently, numerous studies have investigated how inter-limb asymmetry relates to bilateral performance in this population, aiming to enhance understanding and inform strength and conditioning practices (Križaj et al., 2019; Beato et al., 2021; Bishop, Coratella, et al., 2021; Fox et al., 2023). These athletes were required to perform activities demanding maximal lower body strength and power exertion, including jumps, sprints, twists, and rapid and frequent changes of direction. While soccer involves diverse motor skills, strength and power-related measures are deemed the most critical for successful play (Wisløff et al., 2004) and consequently the most frequently evaluated. It has been suggested that asymmetries in strength seem to negatively affect sport-specific performance like change of direction, sprinting, cycling and kicking (Ličen and Kozinc, 2023). Additionally, an increasing number of studies underline the importance of evaluating asymmetry in the realm of injury prevention. It has been shown that inter-limb asymmetry and injury risk might be related (Helme et al., 2021).

Within the body of soccer asymmetry studies, female soccer players participated in just a few of them. Considering the existence of gender differences (Haizlip et al., 2015) it is clear that the lack of asymmetry studies involving female soccer players precludes clear information regarding asymmetry and performance association. The lack of studies on high-performance female athletes limits the ability to apply research findings to the real world and consequently practitioners are often failing to maximize the performance potential of females because applying findings observed on male athletes to female athletes may be erroneous (Emmonds et al., 2019). Nevertheless, few published papers showed asymmetry direction appears highly variable (Bishop et al., 2020), task-specific (Raya-González et al., 2021), and reported a negative association between asymmetry and sprinting and jumping performance (Bishop, Read, et al., 2021). These findings emphasize the need for further research in this field.

Therefore, the present study aim was twofold. Firstly, to investigate the associations between different bilateral and unilateral power/strength-related performance tests and TMG-derived parameters. Secondly, to investigate whether the asymmetries in countermovement jump (CMJ) height, isokinetic strength testing (knee flexion and knee extension at 60°/s and 180°/s) and TMG are associated with bilateral CMJ height, squat jump (SJ) height and speed in elite female soccer players.

## 2 Methods

In the conceptualization phase of the study, we conducted a power analysis using the G*Power (Faul et al., 2007). Based on previous study with similar design (Gil et al., 2015) we expected to find medium correlation between TMG derived parameters and jumping performance (0.5) with power of 0.90 and α = 0.05, two-tailed, which calculated a sample size of 34 participants.

Thirty-five female soccer players (average age: 20 ± 5 years; body height 169.2 ± 6.9 cm; body mass 63.4 ± 7.0 kg; body mass index 22.1 ± 1.4 kg/m^2^) members of the Slovenian National Football Team (Tier 4: Elite level) (McKay et al., 2022) were assessed at the beginning of the new 2021/2022 soccer season. They were advised not to have strenuous workouts for at least 48 h before the assessment, which was monitored by the team staff. Before the initial assessment, a brief meeting was held to explain the study protocol in detail where the written consent of each athlete was obtained. Based on data collection performed by physician, all players were physically healthy, without acute pain, and serious lower limb injuries for at least 1 year. All the participants were informed of the study procedures and provided written informed consent prior to participation. All procedures were approved by an institutional Human Research Ethics Committee (approval decision number: 25:2021) and research was conducted according to Helsinki declaration. Procedures.

A cross-sectional cohort study examined neuromuscular performance and limb differences with corresponding asymmetries in elite female soccer players. Athletes arrived at the testing facility at 8 a.m. and abstained from alcohol and caffeine consumption for at least 24 h prior to testing. All measurements were performed in the following order: basic anthropometry, TMG assessment, SJ, CMJ, speed, agility test, and maximal isometric strength. After the TMG assessment, athletes performed a standardized warm-up protocol by an experienced kinesiologist. For all tests, leg dominance was defined as the kicking leg. All athletes were familiar with the study procedures, having undergone the same battery of tests once per year for a minimum of 2 years before they were recruited for the current investigation. These assessments were conducted as part of regular, periodic measurements at the Institute of Sport, Faculty of Sport, University of Ljubljana.Body mass and height were measured using a stadiometer and scale anthropometer (GPM, Model 101, Zurich, Switzerland) to the nearest of 0.1 cm and 0.05 kg, respectively.

The contractile properties of the individual muscles were assessed by the non-invasive TMG method. We measured knee flexor and extensor muscles bilaterally. Therefore, *m.biceps femoris* (BF) assessment was performed while prone at rest at a knee angle set at 5° knee flexion; whereas the vastus lateralis (VL), vastus medialis (VM), and rectus femoris (RF) were measured while supine at rest at a knee angle set at 30° knee flexion. The well-established methodology was used as previously described (Paravlic et al., 2022). Briefly, following an electrically induced isometric twitch, the radial displacement of the muscle belly was recorded at the skin surface using a sensitive digital displacement sensor (TMG-BMC, Ljubljana, Slovenia). The sensor was set perpendicular to the skin normal plane above the muscle belly as recommended previously (Delagi et al., 2011). The rounded (5-cm diameter) self-adhesive cathode and anode (Axelgaard, Aarhus, Denmark) were set 5 cm distally and 5 cm proximally to the measuring point, on all muscles assessed. Electrical stimulation was applied through a TMG-100 System electro stimulator (TMG-BMC d.o.o., Ljubljana, Slovenia) with a pulse width of 1 ms and an initial amplitude of 20 mA. During each measurement, the amplitude was progressively increased by 20 mA increments until there was no further increase in the amplitude of the TMG response (Dm), which was usually accompanied by the maximal stimuli of 110 mA. Rest periods of 30 s were given between each stimulus in order to minimize the effects of fatigue and potentiation. More detailed testing procedures were previously described elsewhere (Paravlić et al., 2017). From two maximal twitch responses, several TMG parameters were calculated, as follows: delay time (Td) as time from an electrical impulse to 10% of the maximal displacement amplitude (Dm); contraction time (Tc) as time from 10% to 90% of Dm; sustain time (Ts) as time from 50% to 50% of Dm; and half-relaxation time (Tr) as time from 90% to 50% of Dm. Additionally, the index representing the velocity of contraction (Vc) was calculated using Eq. 1 (Loturco et al., 2016):

$$Vc=\frac{Dm}{Td+Tc}\;\left(\text{mm}/\text{ms}\right)$$ (1)

Moreover, the TMG proposed the algorithm for calculating the functional symmetries which was implemented in the current investigation was calculated using Eq. 2.

$$FS=0.1\;x\;\frac{\mathrm{MIN}\left(AVERAGE\left(TdRF;TdVL;TdVM\right);TdBF\right)}{\mathrm{MAX}\left(AVERAGE\left(TdRF;TdVL;TdVM\right);TdBF\right)}+0.8\;x\;\frac{\mathrm{MIN}\left(AVERAGE\left(TcRF;TcVL;TcVM\right);TcBF\right)}{\mathrm{MAX}\left(AVERAGE\left(TcRF;TcVL;TcVM\right);TcBF\right)}+0.1\;x\;\frac{\mathrm{MIN}\left(AVERAGE\left(TrRF;TrVL;TrVM\right);TrBF\right)}{\mathrm{MAX}\left(AVERAGE\left(TrRF;TrVL;TrVM\right);TrBF\right)}\;x\;100$$ (2)

where FS represents a functional symmetry, MIN—the minimum and MAX–the maximum.

A 30-m distance was selected to evaluate sprint performance. Participants performed 2 maximal sprint efforts over the distance of 30 m in an indoor sports hall with a 3-min rest period between trials. The fastest trial was used for further analyses. Players were verbally encouraged to sprint as fast as possible during each trial. Sprint times, and therefore maximal running speed was recorded to 0.001 mm/s accuracy by a laser distance measuring device (Artech LDM 301, Rostock, Germany).

The soccer-specific change of direction test (SSCOD) is recently newly developed agility assessment tool which showed to have high levels of reliability and discriminative validity (Krolo et al., 2020). In brief, a test is performed on the 10 m square field. It starts with the players running with maximal effort to the first cone placed at 1 m distance from the start, following which they need to run at maximal speed in the direction of the second cone placed diagonally (left and right), 3 m away. Then, the players need to kick the ball on the goal with the inside foot and turn back through the starting gate as quickly as possible. All the players were tested on the same pattern and performed two trials for each side, whereas first running in the direction of the cone placed on the left side, and secondly to the right side. The change of direction (i.e., running to the left or right-side placed cone) was predetermined. The time was measured using one pair of electronic timing system sensors (Witty Timing System, MicroGate, Bolzano), with the nearest of 0.1 s, while the average of two trials was taken for further analysis.

Jumping ability was assessed by vertical jump tests, using bilateral force plate (model 9260AA6, Kistler, Winthertur, Switzerland) with Kistler MARS software to record ground reaction force data. Before the actual test a 2–3 warm-up squat jumps, bilateral countermovement jumps (CMJb), and unilateral CMJ (CMJu) with hands placed on the hips were executed, followed by actual maximal jump trials. Test execution was supervised and verbally encouraged by an experienced researcher to improve proficiency in jumping technique. Jumps were repeated after a 60-s rest period, until three valid SJ, CMJb and CMJu jumps were achieved. In total, the number of trials at each jumping condition ranged from three to five. Subjects were instructed and verbally encouraged to maximize their jump height. The depth position during both SJ and CMJ was self-preferred. Jump height was calculated by vertical velocity of the center of mass at take-off. Finally, the jump with the highest jump height was taken for further analysis (Moir, 2008).

Isokinetic strength testing was performed on an isokinetic dynamometer (Biodex 4, Medical System, NY, United States) with the Advantage software. Standard leg attachment was used. The test was performed with the participants in the sitting position, strapped with belts across the chest, pelvis and test leg thigh to minimize body movement and compensation of other muscles. The dynamometer axis of rotation was aligned with the knee’s joint axis of rotations using lateral epicondyle as an anatomic mark. The range of motion was 60° (from 90° to 30° of knee flexion with full knee extension being 0°). Before the testing, each subject performed 5 to ten submaximal knee extension and flexion concentric contractions at 60°/s as part of a specific warm-up. The testing procedure consisted of 5 consecutive knee extension and flexion repetitions at 60°/s and 10 at 180°/s. There was a 2-min break between the velocities. Participants were verbally encouraged by the investigator to perform the test as hard and as fast as possible. Visual feedback was provided throughout the test on the dynamometer monitor. The isokinetic torques for the quadriceps and the hamstrings at each angular velocity (60°/s and 180°/s) were first normalized to the subject’s body mass. We then calculated the isokinetic H:Q ratios at both angular velocities using Eq. 3.

$$H:Q=\frac{peak\;hamstrings\;torque}{peak\;quadriceps\;torque}\times 100$$ (3)

## 3 Inter-limb asymmetry analysis

The mean inter-limb asymmetries were computed for CMJu, isokinetic strength, SSCOD test, and TMG variables by using the Eq. 4:

$$Inter-limb\;asymmetry=100\;–\;\left(\left(\mathrm{MIN}\;value\right)/\left(\mathrm{MAX}\;value\right)\right)\times 100$$ (4)

## 4 Statistical analysis

Statistical analyses were performed using SPSS statistical software (version 27.0, IBM Inc, Chicago, United States). All data are presented as mean ± SD. Descriptive statistics were used to summarize player general characteristics and all outcome measures. Normality was confirmed by visual inspection and using the Shapiro-Wilk test. Inter-limb differences were assessed by the Student t-test. Conversely, a Wilcoxon signed rank test was used for the comparison of variables not following a normal distribution. Hedges’ g effect sizes (ES) with 95% confidence intervals were calculated to show practical differences between legs and were interpreted as: trivial: <0.20, small: 0.20–0.50, moderate: 0.50–0.80, or large: >0.80 (Lovakov and Agadullina, 2021). The Pearson correlation was used to analyze the relationship between the variables of interest that followed a normal distribution. If otherwise, the correlation was analyzed using nonparametric Spearman’s rho. The following thresholds of the correlation coefficient were used to assess magnitude of the relationships analyzed: weak ≤0.35; 0.36≤moderate<0.67; 0.68≤ high <1 (Taylor, 1990). Statistical significance for all analyses was accepted at *p* ≤ 0.05.

## 5 Results

A total of thirty-five players were measured, with the right leg defined as dominant in thirty players. On average, players jumped 29.0 ± 5.1 cm and 30.1 ± 5.8 cm from the CMJb and SJ, respectively, while maximum running speed was 7.7 ± 0.4 m/s.

### 5.1 Inter-limb comparisons

Table 1 shows comparisons between dominant and non-dominant legs with the resulting asymmetries for all measures assessed. A significant difference (*small* ES = 0.47, *p* = 0.008) was observed in the soccer-specific change of direction test, where players took less time to complete the test when they changed direction towards the dominant side. In addition, compared to the non-dominant leg, the dominant leg showed greater values for knee extension torque at 60°/s (small ES = −0.43, *p* = 0.014), peak knee flexion torques at both angular velocities 60°/s (small ES = −0,36, *p* = 0.038) and 180°/s (small ES = −0.47, *p* = 0.009), while the HQ peak torque ratio at 180°/s was lower for the dominant leg (small ES = −0.42, *p* = 0.018). As for the TMG variables, only the contraction time of the VM was shorter on the dominant side (small ES = −0.46, *p* = 0.011). A detailed inter-limb comparison of the CMJ-derived parameters between dominant and non-dominant legs are presented in Supplementary Table S1.

**TABLE 1:** Inter-limb comparison of the soccer-specific change of direction test, countermovement jump, squat jump, isokinetic strength and tensiomyography-derived parameters between dominant and non-dominant legs in elite female soccer players.

Variables	Non-dominant leg		Dominant leg		Inter-limb asymmetries (%)		Inter-limb comparison				
Unilateral tests	Mean	SD	Mean	SD	Mean	SD	t or Z value	*p*-value	Hedges’s g	LCI	UCI
Agility											
Soccer specific COD test (s)	2.67	0.16	2.61	0.14	4.02	2.59	2.838	**0.008**	0.47	0.12	0.82
Strength and power											
CMJ unilateral (cm)	14.1	3.5	14.3	3.3	8.55	5.59	−0.847	0.397	−0.10	−0.43	0.23
Knee extension peak torque at 60°/s (Nm/kg)	2.68	0.29	2.76	0.27	5.55	4.42	−2.586	**0.014**	−0.43	−0.77	−0.09
Knee flexion peak torque at 60°/s (Nm/kg)	1.36	0.19	1.41	0.20	7.36	6.06	−2.159	**0.038**	−0.36	−0.70	−0.02
HQ peak torque ratio at 60°/s (%)	51.33	7.64	51.04	7.33	6.96	5.56	−0.353	0.726	−0.06	−0.39	0.27
Knee extension peak torque at 180°/s (Nm/kg)	1.86	0.23	1.88	0.23	5.76	3.80	−0.622	0.538	−0.10	−0.43	0.23
Knee flexion peak torque at 180°/s (Nm/kg)	0.99	0.18	1.05	0.20	8.19	7.06	−2.788	**0.009**	−0.47	−0.81	−0.12
HQ peak torque ratio at 180°/s (%)	55.92	8.18	53.30	5.73	8.47	6.03	−2.375	**0.018**	−0.42	−0.76	−0.07
TMG parameters											
Biceps Femoris Tc (ms)	28.64	5.22	28.34	4.96	10.43	8.01	−0.033	0.974	0.07	−0.26	0.40
Rectus Femoris Tc (ms)	29.25	4.10	28.52	3.73	8.23	5.93	−1.245	0.213	0.22	−0.11	0.55
Vastus Lateralis Tc (ms)	20.59	1.74	20.86	1.92	6.57	4.49	−1.097	0.272	−0.15	−0.48	0.18
Vastus Medialis Tc (ms)	24.06	2.31	23.08	2.56	7.64	5.06	−2.539	**0.011**	0.46	0.11	0.81
Biceps Femoris Vc (mm/ms)	0.08	0.03	0.08	0.03	46.44	16.16	0.230	0.819	0.04	−0.29	0.37
Rectus Femoris Vc (mm/ms)	0.13	0.04	0.13	0.04	14.24	11.49	−0.974	0.337	−0.16	−0.49	0.17
Vastus Lateralis Vc (mm/ms)	0.12	0.03	0.12	0.02	12.00	6.58	−1.335	0.191	−0.22	−0.55	0.11
Vastus Medialis Vc (mm/ms)	0.14	0.03	0.15	0.02	9.56	6.68	−0.642	0.525	−0.11	−0.44	0.22
Functional Knee symmetry (%)	83.97	9.33	81.08	7.29	8.21	6.67	−1.933	0.053	0.32	−0.02	0.65

CMJ, countermovement jump; HQ, hamstring to quadriceps peak torque; LCI, lower confidence interval; UCI, upper confidence interval. Bold values represent significant difference.

### 5.2 The interrelationship between countermovement jump, squat jump, maximum running speed, and different isokinetic peak torque testing parameters

Table 2 shows the correlations between CMJ, SJ, Maximum running speed on 30°m distance (MRS30), and various isokinetic peak torque test parameters performed with dominant and non-dominant legs. For CMJb, significant correlations were observed for most of the variables studied, ranging from weak (*r* = 0.354, *p* = 0.037, for dominant leg H/Q at 60°/s) to moderate (*r* = 0.482, *p* = 0.005, for dominant knee extension peak torque at 180°/s). For SJ, a significant correlation was also observed, ranging from weak (*r* = 0.345, *p* = 0.042, for dominant knee extension peak torque at 180°/s) to moderate (*r* = 0.532, *p* = 0.001, for non-dominant knee flexion peak torque at 180°/s). Finally, for the maximal running speed, correlations ranged from slightly moderate (*r* = 0.363, *p* = 0.032, for dominant knee extension peak torque at 60°/s) to moderate (*r* = 0.563, *p* < 0.001, for non-dominant knee flexion peak torque at 180°/s).

**TABLE 2:** Correlation between countermovement jump, squat jump, maximum running speed and different isokinetic peak torque testing parameters performed with dominant (Dom) and non-dominant (Ndom) legs in elite female soccer players.

	CMJb	SJ	MRS30 m	CMJu Ndom	CMJu Dom	SSCOD Ndom	SSCOD Dom
*SJ*	**.795** ^**^						
*MRS30m*	**.649** ^**^	**.710** ^**^					
CMJu Ndom	**.837** ^**^	**.673** ^**^	**.654** ^**^				
CMJu Dom	**.776** ^**^	**.646** ^**^	**.583** ^**^	**.901** ^**^			
SSCOD Ndom	−0.33	−0.309	**−.521** ^**^	−0.264	−0.228		
SSCOD Dom	−0.243	**−.360** ^*^	**−.427** ^*^	−0.043	0	**.692** ^**^	
KET60 Ndom	0.213	0.134	0.193	0.272	0.285	−0.251	0.099
KET60 Dom	0.168	0.167	0.089	0.278	**.375** ^*^	−0.106	0.25
KF60 Ndom	**.454** ^**^	**.402** ^*^	0.313	.412^*^	**.422** ^*^	−0.294	−0.176
KF60 Dom	**.467** ^**^	**.493** ^**^	**.363** ^*^	**.498** ^**^	**.531** ^**^	−0.016	0.05
KET180 Ndom	**.448** ^**^	**.399** ^*^	**.524** ^**^	**.546** ^**^	**.463** ^**^	**−.377** ^*^	−0.108
KET180 Dom	**.482** ^**^	**.345** ^*^	**.437** ^**^	**.566** ^**^	**.588** ^**^	−0.329	0.043
KF180 Ndom	**.467** ^**^	**.532** ^**^	**.563** ^**^	**.558** ^**^	**.437** ^**^	**−.458** ^**^	−0.313
KF180 Dom	**.411** ^*^	**.402** ^*^	0.27	**.419** ^*^	**.375** ^*^	−0.293	−0.147
HQr60 Ndom	**.354** ^*^	**.368** ^*^	0.321	0.318	0.256	0.053	−0.142
HQr60 Dom	0.291	0.296	0.187	0.212	0.206	−0.091	−0.267
HQr180 Ndom	0.139	0.225	0.004	0.084	−0.002	−0.092	−0.21
HQr180 Dom	0.234	**.390** ^*^	0.303	0.263	0.176	−0.308	**−.389** ^*^

Dom, dominant leg; Ndom, Non-dominant leg; CMJb, bilateral countermovement jump; CMJu, unilateral countermovement jump; SJ, squat jump; MRS30 m, Maximum running speed on 30 m distance; SSCOD, soccer specific change of direction test; KET60, Knee extension peak torque at 60°/s; KET180, Knee extension peak torque at 180°/s; KF, knee flexion peak torque; HQr, Hamstring to quadriceps peak torque ratio. Bold values represent significant difference.

### 5.3 The interrelationship between countermovement jump, squat jump, maximum running speed, and different tensiomyography-derived parameters

Table 3 and Table 4 shows the correlations between CMJ, SJ, MRS30 and various tensiomyography-derived parameters performed with dominant and non-dominant legs. For CMJb, significant correlations were observed only for a few variables studied, ranging from moderate (*r* = 0.337, *p* = 0.048, for dominant leg BF Vc) to moderate (*r* = 0.537, *p* = 0.001, for non-dominant leg RF Tc). For SJ, a significant correlation was also observed, ranging from weak (*r* = −0.354, *p* = 0.037, for non-dominant leg BF Tc) to moderate (*r* = −0.512, *p* = 0.002, for dominant leg BF Tc). Finally, for MRS30, correlations ranged from weak (*r* = −0.345, *p* = 0.042, for dominant leg VM Tc) to moderate (*r* = −0.530, *p* = 0.001, for non-dominant leg BF Tc). A comprehensive presentation of the association between bilateral CMJ-derived parameters and TMG-derived parameters are presented in Supplementary Figure S1, S2.

**TABLE 3:** Correlation between countermovement jump, squat jump, maximum running speed and different tensiomyography-derived parameters performed on dominant (Dom) and non-dominant (Ndom) legs in elite female soccer players.

	*CMJb*	*SJ*	*MRS30m*	BF Tc Ndom	BF Tc Dom	RF Tc Ndom	RF Tc Dom	VL Tc Ndom	VL Tc Dom	VM Tc Ndom	VM Tc Dom
*SJ*	**.795** ^**^										
*MRS30m*	**.649** ^**^	**.710** ^**^									
BF Tc Ndom	−0.33	**−.354** ^*^	**−.530** ^**^								
BF Tc Dom	**−.526** ^**^	**−.512** ^**^	**−.497** ^**^	**.660** ^**^							
RF Tc Ndom	**−.537** ^**^	**−.387** ^*^	−0.223	**.335** ^*^	0.29						
RF Tc Dom	**−.459** ^**^	**−.425** ^*^	**−.406** ^*^	**.353** ^*^	**.370** ^*^	**.667** ^**^					
VL Tc Ndom	−0.277	−0.105	−0.133	0.243	0.146	**.486** ^**^	**.353** ^*^				
VL Tc Dom	**−.350** ^*^	−0.198	−0.271	**.527** ^**^	**.473** ^**^	**.519** ^**^	**.535** ^**^	**.536** ^**^			
VM Tc Ndom	**−.519** ^**^	**−.439** ^**^	**−.392** ^*^	**.492** ^**^	**.396** ^*^	**.482** ^**^	**.432** ^**^	**.513** ^**^	**.401** ^*^		
VM Tc Dom	**−.487** ^**^	−0.289	**−.345** ^*^	0.291	0.278	0.317	0.243	0.274	0.214	**.637** ^**^	
BF Vc Ndom	0.113	0.115	0.076	0.145	0.087	0.072	0.231	0.017	0.024	0.01	0.039
BF Vc Dom	**.337** ^*^	0.272	0.219	−0.003	−0.008	−0.269	−0.092	−0.2	−0.146	−0.229	0.056
RF Vc Ndom	**.372** ^*^	0.312	0.203	−0.243	−0.058	−0.263	0.017	−0.087	−0.141	−0.311	−0.091
RF Vc Dom	0.321	0.28	0.113	**−.357** ^*^	−0.069	−0.201	0.075	−0.157	−0.004	−0.321	−0.004
VL Vc Ndom	0.328	0.241	0.245	−0.228	−0.225	−0.306	−0.023	0.164	−0.166	−0.087	0.029
VL Vc Dom	0.225	0.124	0.096	−0.184	−0.128	−0.241	0.141	0.006	−0.005	−0.292	−0.074
VM Vc Ndom	0.169	0.226	0.179	−0.207	−0.103	−0.065	0.192	0.129	0.046	−0.262	−0.32
VM Vc Dom	0.101	0.196	0.159	−0.202	−0.174	−0.021	0.194	0.133	0.071	−0.101	−0.038

Dom, dominant leg; Ndom, Non-dominant leg; CMJb, bilateral countermovement jump; SJ, squat jump; MRS30 m, Maximum running speed on 30 m distance; BF, Biceps femoris; RF, Rectus femoris; VL, Vastus Lateralis; VM, Vastus medialis; Tc, Contraction time; Vc, Contraction velocity. Bold values represent significant correlation.

**TABLE 4:** Correlation between countermovement jump, squat jump, maximum running speed and inter-limb asymmetries (a) derived from countermovement jump, agility, isokinetic and tensiomyography testing procedures performed with dominant and non-dominant legs.

	*CMJb*	*SJ*	*MRS30m*	aCMJ	aSSCOD	aKET60	aKFT60	aHQr60	aKET180	aKET180	a HQr180
*SJ*	**.773** ^**^	1									
*MRS30m*	**.664** ^**^	**.705** ^**^	1								
aCMJ	−0.283	−0.094	−0.235	1							
aSSCOD	−0.225	−0.242	−0.047	−0.114	1						
aKET60	−0.174	−0.164	−0.202	−0.004	−0.073	1					
aKFT60	−0.265	−0.052	−0.17	0.308	0.006	0.191	1				
aHQr60	−0.13	0.26	0.191	**.420** ^*^	−0.079	−0.003	**.412** ^*^	1			
aKET180	−0.081	−0.238	−0.247	0.053	0.091	0.287	0.138	−0.113	1		
aKET180	0.122	0.021	−0.069	**.341** ^*^	−0.182	−0.006	0.218	−0.181	0.023	1	
aHQr180	0.063	−0.1	−0.188	0.195	−0.276	0.015	0.002	−0.107	.398^*^	**.549** ^**^	1
aBFTc	−0.056	−0.08	−0.298	0.095	−0.208	−0.08	0.097	−0.082	0	0.206	0.236
aRFTc	−0.216	−0.154	−0.227	0.186	0.183	0.268	0.076	0.102	0.231	−0.088	−0.08
aVLTc	**−.419** ^*^	−0.286	−0.183	0.138	0.191	0.006	0.152	0.031	0.203	−0.107	0.004
aVMTc	0.037	−0.091	0.016	−0.148	−0.221	0.05	−0.152	**−.346** ^*^	0.102	−0.01	0.104
aBFVc	0.069	0.153	0.069	−0.067	0.017	−0.307	−0.01	0.227	−0.208	0.005	0.027
aRFVc	−0.296	−0.272	−0.047	−0.072	0.313	0.033	−0.027	0.113	−0.222	**−.402** ^*^	**−.337** ^*^
aVLVc	−0.177	−0.326	−0.213	0.265	0.191	0.021	0.003	−0.028	−0.003	0.087	0.178
aVMVc	−0.008	−0.029	0.123	0.133	**−.339** ^*^	−0.055	0.06	0.08	−0.002	−0.026	0.203
aFKSTMG	−0.021	−0.094	−0.243	0.147	−0.087	0.174	**.393** ^*^	0.055	0.003	0.093	0.004

CMJb, bilateral countermovement jump; SJ, squat jump; MRS30 m, Maximum running speed on 30 m distance; SSCOD, soccer specific change of direction test; KET60, Knee extension peak torque at 60°/s; KET180, Knee extension peak torque at 180°/s; KFE, knee flexion peak torque; HQr, Hamstring to quadriceps peak torque ratio; BF, Biceps femoris; RF, Rectus femoris; VL, Vastus Lateralis; VM, Vastus medialis; Tc, Contraction time; Vc, Contraction velocity; as, asymmetry. Bold values represent significant correlation.

## 6 Discussion

The present study investigated associations between different bilateral and unilateral performance-related screening tests. Moreover, we addressed the question, whether the inter-limb asymmetries detected by CMJ, isokinetic strength and TMG are associated with bilateral performance assessed by CMJ, SJ and speed in elite female players. At first, we observed significant inter-limb asymmetries in favor of dominant side for peak torque output in both knee flexion (7.4%) and knee extension (5.6%) isokinetic tasks, as well as VM contraction time (7.6%), and soccer specific agility test (4.1%). On the other side, the H:Q peak torque ratio at 180°/s was significantly greater (8.5%) in non-dominant leg. Considering the “10% rule” suggested by Bishop and colleagues (Bishop, Coratella, et al., 2021), we may conclude asymmetries we found were in line with previous studies (Ruas et al., 2015; Loturco et al., 2019) supporting the fact that inter-limb asymmetries in football players of both sexes, are smaller than the borderline values proposed (Ruas et al., 2015). Interestingly, although similar asymmetry levels were observed in previous studies recruiting male soccer players (Daneshjoo et al., 2013; Menzel et al., 2013), we found that the preferred kicking leg outperformed contralateral leg in both knee extension and flexion strength task, which is opposite to aforementioned studies recruiting male soccer players (Daneshjoo et al., 2013; Menzel et al., 2013). This phenomenon has been observed elsewhere in female athletes (Risberg et al., 2018; Eustace et al., 2019) and identified as a potential risk factor for a more frequent occurrence of anterior cruciate ligament injuries in female soccer players (Brophy et al., 2010). The mechanics behind this adaptation has been previously described and include factors like anthropometric, physiological and gameplay differences between male and female soccer (Pedersen et al., 2019).

Another interesting observation in current study were variations in force-time strategies between the dominant and non-dominant legs, despite achieving similar jump performance (Supplementary Table S1). Specifically, the non-dominant leg exerted a higher maximal force relative to body weight (183.9% BW vs. 179.3% BW), greater average force (916.1 N vs. 900.6 N), and spent less time in the push-off phase (0.34 s vs. 0.36 s) than the dominant leg. This suggests that athletes may have reached a greater depth position during the CMJ with the non-dominant leg. This could be attributed to biomechanical and neuromuscular differences between the dominant and non-dominant legs which may have importance in players profiling and identification of athletes who were at risk of sustaining injury (Bishop, Read, et al., 2021; Mitchell et al., 2021; Roso-Moliner et al., 2023). A recent study (Mitchell et al., 2021) reported compromised performance in single-leg CMJ among athletes with a history of injury. This was characterized by significantly reduced eccentric and concentric peak force, lower rates of force development, and a deeper countermovement. Conversly, the players recruited in our study were physically healthy, without acute pain, and free from serious lower limb injuries for at least 1 year before recruitment. Thus, the observed differences in time-force characteristics may result from previous injuries that have been repaired (happened more than a year before the recruitment), or they may represent a risk factor for future injuries, which still warrants further investigation.

Further on, different bilateral and unilateral screening tests showed statistically significant and slightly moderate to large correlation. In detail, a weak to moderate correlation was found between highest height jumped and isokinetic variables, and slightly moderate to large correlation between highest height jumped and TMG-derived parameters. We also found moderate correlation between VL contraction time asymmetry and bilateral CMJ performance. These results support the fact that the isokinetic dynamometry test and the TMG, as well as the corresponding inter-limb asymmetries, are highly variable in indicating performance related to bilateral jumping ability and running speed-related tasks. This has also been shown in the recent study conducted by Loturco et al. (2019). Loturco and colleagues (Loturco et al., 2019) found that CMJ and SJ asymmetry index does not correlate or impair maximal running speed and bilaterally assessed jumping performance in professional female soccer players. The later study also found nonsignificant to slightly moderate significant correlation coefficients between performance tests and bilateral and unilateral vertical jump variables. However, no significant correlations were found between unilateral vertical jump asymmetries and other performance tests were found.

The overall significant, albeit inconsistent, correlations between the diverse performance scores obtained highlight the previously recognized necessity for a multifaceted and thorough diagnostic strategy in female soccer (Loturco et al., 2018; 2019) aiming at examining multiple performance aspects. These are further supported by the nonsignificant asymmetry-performance relationship indicating even the asymmetry evaluation requires multiple approaches. For example, some conflicting evidence found previously illustrated this issue. Namely, Loturco and colleagues (Loturco et al., 2018) found better performances in SJ and CMJ tests were associated with higher asymmetry levels whilst Bishop and colleagues (Bishop, Read, et al., 2021) found greater asymmetries being associated with reduced jump performance. Therefore, it is still unclear whether certain asymmetry parameters inevitably influence bilateral performance evaluated by maximal running speed and jumping performance. Considering the previously stated and the fact that injury types (Cross et al., 2013) and mechanisms (Brophy et al., 2010) differ between female and male athletes, there is need for the development of unique testing procedures sensitive enough for multidimensional purposes, including performance profiling, neuromuscular fatigue and performance monitoring, injury prevention and rehabilitation testing in female soccer players and female athletes in general.

## 7 Conclusion

The present study added a new knowledge on performance assessment in female soccer players. First, we found significant differences in several performance measures of interest when comparing dominant and non-dominant legs, that may have an important indices for training program optimization and prevention of future injuries. Second, the overall significant, albeit inconsistent, correlations between the diverse performance scores obtained highlight the necessity for a multifaceted and thorough diagnostic strategy in female soccer players. From the results observed it is difficult to choose a single test as a unique screening tool for performance profiling. We believe, it would be valuable to consider the implications of these findings on performance, injury risk, and training strategies, providing a more comprehensive understanding of the biomechanics involved in unilateral jumping movements.

## 8 Perspectives

The findings of the current study have implications for the training and assessment of elite female soccer players. A comprehensive diagnostic strategy that considers a variety of performance measures, including bilateral and unilateral assessments as well as TMG parameters, is essential for a thorough understanding of an athlete’s capabilities. For future perspective, this study suggests that a more extensive longitudinal evaluation with a larger and diverse sample of female soccer players could help establish more robust and generalizable relationships between these performance-related measures. Additionally, examining how these associations evolve over time or in response to specific training interventions may aid in tailoring individualized training programs to enhance performance and reduce the risk of injury.

## Data Availability

The raw data supporting the conclusion of this article will be made available by the authors, without undue reservation.
