# Supplementary material for: The association between unilateral and bilateral performance-related measures in elite female soccer players: a multifaceted investigation
Source: Front Physiol. 2024 Jun 17;15:1298159. doi: 10.3389/fphys.2024.1298159 (PMC11215189; doi:10.3389/fphys.2024.1298159)
Supplement: Supplementary file 1 [file DataSheet1.docx]

**SUPPLEMENTARY MATERIAL**

Table 1. Inter-limb comparison of the CMJ-derived parameters between dominant and non-dominant legs in elite female soccer players.

|  |  |  | Dominant leg | | Non-dominant leg | |  |  |  |  |  |  |
| --- | --- | --- | --- | --- | --- | --- | --- | --- | --- | --- | --- | --- |
|  |  |  | Mean | SD | Mean | SD | MD | t-value | p value | ES | LLCI 95% | ULCI 95% |
| Jump Height from Take Off V [m] | | | 0.14 | 0.03 | 0.14 | 0.04 | 0.002 | 0.90 | 0.376 | 0.15 | -0.18 | 0.48 |
| Jump Height from Flight T [m] | | | 0.15 | 0.03 | 0.15 | 0.03 | -0.001 | -0.45 | 0.658 | -0.07 | -0.40 | 0.25 |
| Start interval relative F [%BW] | | | 171.70 | 14.03 | 175.11 | 16.37 | -3.409 | -1.32 | 0.196 | -0.22 | -0.55 | 0.11 |
| Start interval RFD [N/kg/s] 50 ms | | | 8995.27 | 2113.68 | 9379.19 | 2215.57 | -383.922 | -1.16 | 0.254 | -0.19 | -0.52 | 0.14 |
| Start interval relative P [W/kg] | | | 3.08 | 0.82 | 3.30 | 1.00 | -0.221 | -1.43 | 0.163 | -0.24 | -0.57 | 0.10 |
| Relative maximal F [%BW] | | | 179.29 | 12.89 | 183.93 | 15.41 | -4.638 | -2.17 | **0.037** | -0.36 | -0.70 | -0.02 |
| Relative maximal F during Counter Movement [%BW] | | | 172.48 | 15.53 | 176.49 | 15.60 | -4.012 | -1.60 | 0.119 | -0.27 | -0.60 | 0.07 |
| Relative maximal F during Push Off [%BW] | | | 178.94 | 12.72 | 183.27 | 15.30 | -4.332 | -2.01 | 0.053 | -0.34 | -0.67 | 0.00 |
| Relative maximal P [W/kg] | | | 26.80 | 3.36 | 26.94 | 3.49 | -0.140 | -0.48 | 0.633 | -0.08 | -0.41 | 0.25 |
| Vertical Take Off V [m/s] | | | 1.70 | 0.17 | 1.68 | 0.19 | 0.018 | 1.18 | 0.246 | 0.20 | -0.14 | 0.53 |
| Average P [W] | |  | 902.45 | 131.39 | 908.88 | 134.56 | -6.432 | -0.46 | 0.650 | -0.08 | -0.41 | 0.25 |
| Average F [N] | |  | 900.56 | 100.80 | 916.08 | 102.17 | -15.524 | -2.11 | **0.043** | -0.35 | -0.69 | -0.01 |
| Average V [m/s] | |  | 1.08 | 0.07 | 1.08 | 0.09 | 0.004 | 0.36 | 0.719 | 0.06 | -0.27 | 0.39 |
| Jump T [s] | |  | 0.89 | 0.11 | 0.87 | 0.14 | 0.020 | 0.99 | 0.328 | 0.17 | -0.17 | 0.50 |
| Counter Movement T [s] | | | 0.53 | 0.08 | 0.53 | 0.10 | -0.001 | -0.08 | 0.933 | -0.01 | -0.34 | 0.31 |
| Push Off T [s] | |  | 0.36 | 0.06 | 0.34 | 0.05 | 0.022 | 3.01 | **0.005** | 0.50 | 0.15 | 0.85 |
| Flight T [s] | |  | 0.35 | 0.03 | 0.34 | 0.04 | 0.004 | 1.18 | 0.248 | 0.20 | -0.14 | 0.53 |
| Braking T [s] | |  | 0.33 | 0.06 | 0.30 | 0.07 | 0.021 | 1.67 | 0.104 | 0.28 | -0.06 | 0.61 |
| Push Off FI [Ns] | |  | 102.28 | 12.67 | 100.84 | 12.39 | 1.440 | 1.56 | 0.128 | 0.26 | -0.07 | 0.59 |
| Eccentric Deceleration Phase FI [Ns] | | | 51.76 | 15.92 | 53.55 | 14.27 | -1.784 | -0.93 | 0.360 | -0.16 | -0.48 | 0.18 |
| Reactive strength index modified | | | 0.16 | 0.04 | 0.16 | 0.04 | -0.002 | -0.54 | 0.591 | -0.09 | -0.42 | 0.24 |
| LLCI – lower limit of confidence interval; ULCI – upper limit of confidence interval; V – velocity, T – time; BW – body weight; FI – force impulse; Bold values – significant difference | | | | | | | | | | | | |

Figure 1. Correlation (Pearson’s) between bilateral countermovement jump – derived parameters and different tensiomyography-derived parameters performed on dominant (Dom) and non-dominant (Ndom) legs in elite female soccer players.

Figure 2. Correlation (Spearman's rho) between bilateral countermovement jump – derived parameters and different tensiomyography-derived parameters performed on dominant (Dom) and non-dominant (Ndom) legs in elite female soccer players.
